# Rehabilitation for degenerative cervical myelopathy: systematic review and scoping review of UK patient information

**DOI:** 10.1038/s41393-025-01110-z

**Published:** 2025-07-23

**Authors:** Toby O. Smith, Christopher Newton, Ayshea Farrell, Jithy Boby, Jonathan Dove, Fiona Dove, Kelly Turner, Benjamin M. Davies

**Affiliations:** 1 Warwick Medical School, University of Warwick, Coventry, UK; 2 Institute for Applied & Translational Technologies in Surgery, University Hospitals Coventry and Warwickshire NHS Trust, Coventry, UK; 3 Physiotherapy Department, University Hospitals Coventry and Warwickshire NHS Foundation Trust, Coventry, UK; 4 School of Health and Care, University of Coventry, Coventry, UK; 5 Physiotherapy Department, University Hospitals Birmingham, Birmingham, UK; 6 Department of Neurosurgery, University of Cambridge, Cambridge, UK

**Keywords:** Spinal cord injury, Patient education

## Abstract

**Study Design:**

Systematic Literature Review & Patient-Information Scoping Review

**Objectives:**

To assess the evidence on prehabilitation and post-operative rehabilitation interventions for people undergoing surgery for degenerative cervical myelopathy (DCM) and to determine what publicly accessible information is provided to patients from the NHS surrounding DCM surgery.

**Methods:**

A systematic literature review was searched from inception to 19 May 2025. Studies reporting pain, function, disability or quality of life for prehabilitation or post-operative rehabilitation interventions for people undergoing DCM surgery were eligible. Downs and Black appraisal tool was used to assess study quality. Data were analysed in a narrative analysis. Secondary, a review of UK NHS Patient Information Documents (PID) was searched using a Google platform assessment. PID reporting prehabilitation or post-operative information for people awaiting DCM surgery were included. The type of information being provided were extracted and descriptive statistics were used to report frequency of information provision.

**Results:**

From 5218 screened studies, six studies (n = 685) met the eligibility criteria. The evidence was low to moderate in quality. Rehabilitation offered demonstrated improved clinical outcomes but there was limited evidence compared to non-rehabilitation or superiority between different rehabilitation strategies. The PID review identified 38 documents. This indicates education and guidance is commonly offered on returning to work (68%), driving (76%) and normal activities of daily living (63%).

**Conclusion:**

There remains uncertainty on what should be and is offered to patients with DCM in respect to prehabilitation or post-operative rehabilitation. Robust clinical trial evidence on rehabilitation approaches for this population is needed.

**Registration:**

PROSPERO (CRD42024604184).

## Introduction

Degenerative cervical myelopathy (DCM) is characterised by subacute or chronic compression of the cervical spinal cord which results in gradual and progressive neurologic dysfunction [1]. People with DCM often report pain and numbness in limbs, poor coordination, imbalance and bladder problems. The estimated prevalence of DCM is 2.3% worldwide, although most is undiagnosed [2]. Since DCM is largely degenerative in nature, the prevalence is predicted to increase with an ageing population.

DCM is often managed surgically for those with moderate to severe, or progressive symptoms, to stop disease progression. However, response to treatment is dependent on the degree of preoperative functional impairment and symptom duration [3, 4]. Maximal recovery occurs at around six to 12 months. Long-term functional deficits are common. These can include falls and reduced mobility, incontinence, depression, difficulties with self-care and pain, leaving individuals with life-long disabilities. Dependency on analgesia and mental health services remain high [5, 6].

Prehabilitation and post-operative rehabilitation can be provided to people who undergo elective surgery. Both prehabilitation and rehabilitation are aimed to improve outcomes following surgical interventions, thereby addressing such long-term deficits. Prehabilitation programmes have repeatedly demonstrated in other populations to improve outcomes such as function, preparedness for surgery and reduced hospital length of stay [7, 8]. Similarly, evidence from other populations, notably traumatic spinal cord and brain injury, and stroke, suggests that rehabilitation, and particularly early rehabilitation following injury or surgery, can improve outcomes for people with central nervous system trauma [9–12]. In these areas rehabilitation aims to maximise function, enhance well-being and enable a satisfactory independence in activities of daily living and return to social or occupational pursuits.

These wider success stories underpin a global consensus on the opportunity for rehabilitation in DCM, that led to its selection amongst the top ten research priorities [2]. However, the current state of evidence is unknown [13]. A previous systematic review focused on post-operative rehabilitation and only considered evidence published to 2017 [14]. There is also uncertainty on what patients are currently advised in relation to pre-operative and post-operative rehabilitation in practice. Therefore, the purpose of this study was to answer these questions; to examine the currently available literature on both prehabilitation and rehabilitation for surgery for DCM, and to determine what current information provision is for people awaiting these operations by reviewing information leaflets used across the UK health service (NHS).

## Methods

### Phase 1: systematic review

This systematic review was reported to satisfy the Preferred Reporting Items for Systematic Review and Meta-Analyses (PRISMA) guidelines [15]. The protocol was registered in the PROSPERO international prospective register of systematic reviews (Ref: CRD42024604184).

#### Eligibility criteria

We included studies (randomised (RCT) and non-randomised controlled trials (nRCT)) assessing the effect of prehabilitation or post-operative rehabilitation for people undergoing surgery for DCM. Prehabilitation was defined as an intervention provided any time before surgery once being scheduled/listed for an operation for DCM. This included, at a minimum, education and knowledge sharing AND some form of exercise or physical activity prescription. We did not define what post-operative rehabilitation consisted of nor when it should start or end post-operatively. We included studies which reported clinical outcomes including: physical function, pain, health-related quality of life and complications. We excluded cohorts where patients were undergoing cervical surgery for other neurological impairments such as cervical radiculopathy or stabilisation for trauma. Eligible papers were required to explicitly state that some or all participants had an indication for surgery of DCM or equivalent nomenclature for this disease. Comparator interventions could have either been no prehabilitation or post-operative rehabilitation, or a comparator intervention or programme.

We did not place a date or country restriction on the eligibility criteria. We included systematic reviews and meta-analyses to scrutinise their reference lists for potentially eligible studies.

#### Search strategy

The literature search was performed by one reviewer (TS). This was of the published literature databases: EMBASE, MEDLINE, CINAHL and PubMed. We accessed unpublished or ongoing study data from registries including the WHO International Clinical Trial Registry and ClinicalTrials.gov. The electronic search strategy adopted for EMBASE is presented as Supplementary File 1. This search strategy was adapted for each database. Searches were performed from database inception to 19 May 2025.

A forward-citation search was performed for all included studies using the Scopus database. Secondly, a backward-citation search was conducted through a review of all included study reference lists.

#### Study identification

All titles and abstracts from the search strategy results were independently reviewed by one reviewer (TS) and verified by a second reviewer (CN). The full-text papers for all potentially eligible studies were reviewed independently by each reviewer to ascertain final eligibility. Any disagreements between reviewers were resolved through discussion.

#### Data extraction

Data were extracted onto a pre-defined data extraction form by one reviewer (TS) and verified by a second (CN). Disagreements in data extraction between reviewers were resolved through discussion.

Where the same study was reported across two or more papers, these were classified as a single study to avoid multiple counting.

Data extracted included: country of origin, year of study conduct, number and characteristics of participants, participant age and gender, previous surgery, duration of symptoms, baseline functional status, prehabilitation intervention, post-surgical rehabilitation intervention and clinical outcomes at each time-point. For each intervention, details on what was performed, when, by whom, any form of tailoring and how, was captured.

#### Outcome measures and endpoints

The primary outcome was physical function at medium-term assessment (three months to 12 months post-operatively). Secondary outcomes reflected the RECODE-DCM core outcome set [16] and included pain, quality of life and adverse events. Outcomes were categorised as short-term (zero weeks to three months) post-operatively, medium-term (three months to 12 months) and longer-term (over 12 months).

#### Critical appraisal

One reviewer (TS) critically appraised each included study using the Downs and Black Critical Appraisal Tool [17] for nRCTs. This was then verified by a second reviewer (CN). Disagreements between reviewers in scoring items were resolved through discussion.

#### Data analysis

Data extraction tables were reviewed for study heterogeneity. Through this, between-study variability in participant characteristics, interventions and study design were assessed. Heterogeneity in study interventions occurred which precluded the ability to undertake meta-analysis. Accordingly, a narrative analysis of the literature was undertaken [18].

### Phase 2: scoping review of UK NHS patient information

#### Search strategy

One reviewer (TS) performed an electronic search of Google for publicly-accessible patient information document (PID) from websites of UK NHS Trusts reporting pre- or post-operative information for people awaiting DCM surgery. All searches were performed 01 November 2024. The search terms are presented in Supplementary File 2.

#### Eligibility criteria

We included PID provided from UK NHS hospitals, describing information on pre-operative preparedness for DCM surgery and/or guidance offered for post-operative recovery and rehabilitation. A restriction to the UK was a pragmatic, limiting the boundaries of the search to minimise the risk resources were overlooked, whilst ensuring the results remain representative. The NHS provides healthcare free of charge to all UK citizens within an integrated healthcare system, whereby spinal surgery is provided by 42 centres. Included PID may therefore have provided information on pre-operative exercises, activities and lifestyle modifications, information on the operation and what to expect during the in-patient stay, and post-operative recovery and rehabilitation recommendations. We excluded guidance provided from non-UK NHS settings including private or charity health providers. We excluded PID which offered non-operative guidance and information (i.e. non-surgical management). We excluded PID which specifically focused on surgery for cervical radiculopathy with no reference to DCM or equivalent nomenclature.

Google searches were continued until one full search page returned no relevant PID. This follows previously adopted methods [19]. We compared and amalgamated the results from each of the searches performed.

#### Data extraction

One reviewer (TS) extracted data from each included PID onto to pre-defined data extraction table. These data were verified by a second reviewer (either CN, KT, JB, FD, JD, AF). Any disagreements in data extraction were resolved through discussion.

#### Statistical analysis

We used descriptive statistics to examine the frequency of elements which were reported in PIDs such as percentage reporting pre-surgical expectations, advice of pre-operative exercise or post-operative return to work recommendations. All analyses were performed on SPSS (IBM SPSS, Version 29.2.0 (20)).

## Results

### Phase 1: systematic review

#### Search strategy results

**Fig. 1:**
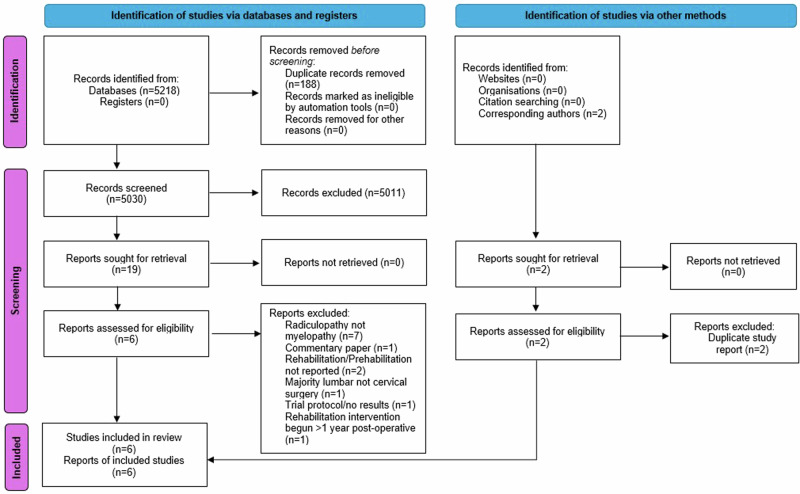
PRISMA flow-chart summarising the results of the search strategy.

The search strategy results are presented in Fig. 1. In total, 5218 studies were identified from the search. Of these 19 were deemed potentially eligible. Following full-text review, six studies were eligible and included in the analysis.

#### Characteristics of included studies

Three studies were retrospective observational studies [20–22], two were prospective comparative observational studies [23, 24], whilst one study was a randomised case-controlled trial [25]. Two studies were performed in the USA [21, 22], with single studies from Israel [20], Taiwan [23], Iran [25] and Japan [24].

In total, 596 participants (362 male; 234 female) were included. Mean age was 60.0 years (standard deviation (SD): 8.0). DCM was the predominant pathology in all studies although mixtures of DCM with radiculopathy cohorts were included in three studies [20–22]. Cheng et al. [23] compared clinical outcomes between a DCM and healthy, non-pathological cohort. All but Catz et al. [20] stated the surgical interventions participants underwent some form of decompression with or without fixation. Fifty-five participants in Lorentz et al. [22] cohort receiving cervical disc replacement surgery.

The studies of rehabilitation interventions are summarised in Table 1. No prehabilitation interventions were identified. All post-operative rehabilitation interventions varied in relation to their length, composition and dosage. These ranged from assessing an exercise-based outpatient rehabilitation programme [22, 24], goal-setting, psychological and physical rehabilitation focusing on social function [24], in-patient rehabilitation programme [20], outpatient physiotherapy programme versus repetitive transcranial magnetic stimulation (rTMS) [25] and outpatient physiotherapy and a telephone-based home exercise programme delivered by a physical therapist [21].

**Table 1:** Data extraction for systematic review included studies.

Study	Design	Country of Origin	N	Age (mean; SD)	Sex	Diagnosis	Surgical Intervention	Rehabilitation Intervention	Follow-up Period
Catz [20]	Retrospective observational	Israel	116	60.6 (12.0)	M: 85 F: 31	Surgery for cervical spinal degenerative changes. Majority DCM.	Not stated	Comprehensive in-patient rehabilitation – multidisciplinary and goal-setting approach. No further details. LOS in rehab 72 days (40)	72 days (SD 40)
Cheng [23]	Prospective comparative observational	Taiwan	29	DCM: 64.0 (5.3) Healthy: 67.4 (5.9)	M: 15 F: 14	Health: 14 DCM: 15	Cervical decompression	Experimental: Perturbation-based balance training (2, 1 hr sessions weekly for 4 weeks (8 sessions)) using perturbation treadmill received by DCM group (n = 15). Comparator: Same perturbation-based training as experimental group but for healthy comparator group (n = 14)	4 weeks
Coronado [21]	Retrospective observational	USA	8	53.4 (14.9)	M: 3 F: 5	Cervical radiculopathy (n = 1), myeloradiculopathy (n = 5), or myelopathy (n = 2)	ACDF	Telephone support home exercise programme in first 6 weeks post-operatively. Progresses in 3 phases every 2 weeks. Weekly phone-calls by physical therapists to promote adherence, progress exercise prescription and increase motivation. Intervention included daily walking, deep breathing, distraction techniques, cervical and upper body ROM and strengthening exercises	6 months
Farrokhi [25]	Randomised case-controlled trial	Iran	52	Overall: 57.3 (11.3) PT (54.08(14.78) PT +rTMS (60.54 (6.55)	M: 40 F: 12	Cervical spine myelopathy (n = 52)	Posterior cervical decompression through laminectomy +/- fusion	Experimental: rTMS (n = 26) + physiotherapy. Interventions begun <6 months post-operatively. rTMS = 2 week treatment (rTMS system was set on 10 Hz frequency (90% rTMs), 1800 pulses in 90 trials (900 pulses [45 trials] were delivered to the right motor cortex [position C4] and 900 pulses [45 trials] were delivered to the left motor cortex [position C3]), and 15-min duration (each trial includes 20 pulses in 2 s and 8 s intertrain interval). This process was repeated for five sessions in five consecutive days). Physiotherapy (5 × 30 min sessions per week for two weeks) consisting of massage; walking; weight transfer; strengthen and stretch the muscles of the neck, shoulder, and upper extremities; and range of motion exercises for upper and lower extremities. Comparator: physiotherapy alone (n = 26) as described in the experimental arm.	2 weeks
Lorentz [22]	Retrospective observational	USA	220	PT: 54.1 No PT: 49.8	M: 125 F: 95	Implied DCM and radiculopathy	ACDF (n = 165) CDR: 55	Experimental: Formal physical therapy: details not provided Comparator: No formal physical therapy.	24 months
Tamai [24]	Prospective comparative observational	Japan	171	UC: 73.5 (7.0) MA: 72.2 (6.9)	M: 94 F: 77	DCM	Open door laminoplasty	Experimental: Multidisciplinary Approach to Improve Social Functioning (n = 31) consisting of patient- orientated rehabilitation with increased clinical psychology input. Fortnightly meetings for 6 months. Same intensity/frequency as usual care Comparator: Usual Care (n = 140): Weekly rehabilitation for 6 months to improve motor function and activities of daily living.	12 months

*ACDF* Anterior cervical decompression and fusion; *CDR* cervical disc replacement; *DCM* degenerative cervical myopathy; *f* female; *hrs* hours; *LOS* length of stay; *m* male; *MA* multi-disciplinary approach; *n* number of participants; *PT* physiotherapy; *rTMS* repetitive transcranial magnetic stimulation; *SD* standard deviation; *UC* usual care; USA – United States of America; short-term (zero weeks post-operatively to three months), medium-term (three months to 12 months) and longer-term (over 12 months).

#### Methodological quality

A summary of the findings of the critical appraisal assessment is presented in Table 2. The evidence ranged from poor to moderate quality. No RCTs were identified, although Farrokhi et al. [25] randomised their cohort in a case-controlled intervention study. Recurrent weaknesses in the evidence were poorly describing cohort characteristics, particularly with regards to pre-surgical disability and presentation, surgical interventions undertaken (all but Coronado et al. [21]) and not adjusting for potential confounding variables on outcome (all but Coronado et al. [21] and Tamai et al. [24]). Interventions investigated were poorly reported in all studies with insufficient information offered to explain potential tailoring or intervention prescription to participants. Furthermore, all studies were either small or underpowered, not specifying an a priori sample size calculation. No studies blinded participants, assessors or any members of the research team to group allocation/intervention exposure.

**Table 2:** Summary of the Downs and Black appraisal results.

	Catz [20]	Cheng [23]	Coronado [21]	Farrokhi [25]	Lorentz [22]	Tamai [24]
Hypothesis/aim/objective clearly described	1	1	1	1	1	1
Main outcomes in Introduction or Methods	1	1	1	1	1	1
Patient characteristics clearly described	0	0	1	0	0	0
Interventions of interest clearly described	1	1	1	1	0	1
Principal confounders clearly described	0	0	1	0	0	1
Main findings clearly described	1	1	1	1	0	1
Estimates of random variability provided for main outcomes	1	1	0	1	0	1
All adverse events of intervention reported	0	0	1	0	0	0
Characteristics of patients lost to follow-up described	0	0	1	0	0	1
Probability values reported for main outcomes	1	1	0	1	1	1
Source of funding described	1	1	1	0	0	1
Subjects asked to participate were representative of source population	1	0	1	1	0	1
Subjects prepared to participate were representative of source population	1	0	1	1	0	1
Location and delivery of study treatment was representative of source population	1	1	1	1	1	1
Study participants blinded to treatment	0	0	0	0	0	0
Blinded outcome assessment	0	0	0	0	0	0
Any data dredging clearly described	0	0	0	0	0	0
Analyses adjusted for differing lengths of follow-up	0	0	1	1	0	0
Approximate statistical tests performed	1	1	1	1	1	1
Compliance with interventions was reliable	0	0	1	0	0	0
Outcome measures were reliable and valid	0	1	1	1	1	1
All participants recruited from the same source population	1	1	1	1	1	1
All participants recruited over the same time period	1	1	1	1	1	0
Participants randomised to treatment(s)	0	0	0	1	0	0
Allocation of treatment concealed from investigators and participants	0	0	0	0	0	0
Adequate adjustment for confounding	0	0	0	0	0	0
Losses to follow-up taken into account	0	0	0	0	0	0
Sufficient power to detect treatment effect at significance level of 0.05	0	0	0	0	0	0
**TOTAL SCORE**	13	13	18	15	9	15

#### Comparison 1: comprehensive rehabilitation programme

Three studies tested a comprehensive physiotherapy programme [20, 22, 24]. Catz et al. [20] assessed this when delivered as an inpatient programme, Lorentz et al. [22] assessed as an outpatient programme, whilst Tamai et al. [24] assessed weekly rehabilitation with a focus on social functioning in recovery.

Both Lorentz et al. [22] and Tamai et al. [24] reported physical function outcomes measured at mid-term. Lorentz et al. [22] reported no significant difference in PROMIS-physical function outcomes for those who received formal out-patient physiotherapy compared to those who did not at six-month (p = 0.37) and 12-month (p = 0.44) assessment. Greater improvement in physical function was demonstrated at 12 months for those who received rehabilitation with focus on social functional rehabilitation compared to conventional physiotherapy when measured using the modified Japanese Orthopaedic Association score (mJOA) (p = 0.04) and JOACMEQ-upper extremity function (p < 0.01) and to a clinical meaningful difference (69.1 vs 57.6) which did not reach statistical significance (p = 0.06) for JOACMEQ-lower extremity function [24].

Tamai et al. [24] reported no difference at 12-month follow-up between those who received conventional rehabilitation or rehabilitation with a focus on social functioning for disability when assessed with the Neck Disability Index (NDI) (18.6 versus 21.0; p = 0.342).

Catz et al. [20] reported significant improvements in pre- to post-rehabilitation treatment for physical function by a mean of 72 days in-patient rehabilitation when assessed using the American Spinal Injury Association motor score (AMD) (Mean Difference (MD): 10.1; SD: 12.7; p < 0.001) and Spinal Cord Independence Measure third version (SCIM III) (MD: 22.0; SD: 17.8; p < 0.001).

Two studies assessed pain outcomes [22, 24]. Tamai et al. [24] assessed visual analogue scale (VAS) neck pain and VAS arm pain at 12 months. Neither measure was reported to differ between those who received conventional rehabilitation or rehabilitation with a bias on social functioning. Lorentz et al. [22] assessed pain intensity and pain interference with the PROMIS instruments. They reported no statistically or clinically meaningful difference between individuals who received formal versus no formal physiotherapy following surgery (pain intensity: p > 0.19; pain interference (p > 0.21) [22].

Only Tamai et al. [24] assessed quality of life outcomes, reported at 12 months. They reported no difference in EQ-5D-5L or JOACMEQ-QOL measured between those who received the conventional rehabilitation or rehabilitation with a focus on social functioning (p > 0.15) [24].

No data were presented for adverse events.

#### Comparison 2: exercise-based programme

Two studies assessed exercise-based rehabilitation programmes [21, 23]. Cheng et al. [23] assessed a perturbation-based balance training programme delivered over four weeks. Coronado et al. [21] assessed a telephone-support home exercise programme delivered in the first six weeks post-Anterior Cervical Decompression and Fusion (ACDF).

Neither study presented data on the review’s primary outcome. Cheng et al. [23] reported four-week JOACMEQ-LEF pre- to post-intervention data, reporting no difference over time. However, Timed Up and Go (TUG) test reported a significant improvement by three seconds, four weeks post-treatment (12 s versus 9 s; p < 0.05) [23].

Short-term disability data was reported by both studies. There was no significant difference reported by Cheng et al. [23] at four weeks for NDI (p = 0.30) or VAS disability measurement (p = 1.00) but reported a mean difference of 14.0 (SD: 78) at six weeks by Coronado et al. [21]. This difference maintained when measured at six months (MD: 15.9; SD: 5.6).

Coronado et al. [21] measured numerical rating scale (NRS) pain scores. They reported clinical meaningful differences in pre- to post-treatment at six-week follow-up for neck pain (MD: 4; SD: 11) and arm pain (MD: 4.4; SD: 2.6). This maintained at six months for both neck pain (MD: 4; SD: 1.9) and arm pain (MD: 3.9; SD: 1.5).

Coronado et al. [21] reported complications over their six-month follow-up period. They reported no serious adverse events including no case of non-union, following their telephone-supported home exercise programme.

No data were presented for quality of life.

#### Comparison 3: transcranial magnetic stimulation

One study compared clinical outcomes of rTMS with physiotherapy versus physiotherapy alone. Whilst previously researched in other populations including stroke [26], this is currently infrequently used in routine clinical practice following spinal surgery. Farrokhi et al. [25] reported physical function outcome at two weeks (immediately post-treatment). They reported that both those who received rTMS with physiotherapy and those who received physiotherapy alone demonstrated significant improvements in functional outcomes when assessed by the American Spinal Injury Association (ASIA) total motor scores (p < 0.01), ASIA lower extremity score (p < 0.01), mJOA (p = 0.001), Ashworth scale (p = 0.003) and Nurick scale (p = 0.001). Between-group differences were not assessed by the specific outcomes but as ‘recovery rate’ [25]. The authors reported significantly greater recovery rate for those who received rTMS with physiotherapy versus physiotherapy alone for ASIA total motor scores (p < 0.05), mJOA (p = 0.002), Ashworth scale (p = 0.021) and Nurick scale (p = 0.006) [25]. However, the post-treatment follow-up period (two weeks) should be acknowledged as a major limitation and insufficient when considering the efficacy of this rehabilitation intervention.

No data were presented for pain, quality of life or adverse events.

### Phase 2: UK NHS patient information

In total, 38 PIDs were identified. The results of these are presented in Table 3. The majority were published between 2022 to 2024 (68.4%) with 90% deriving from England. Whilst all were cervical spine specific with a focus on degenerative cervical spine surgery, only two documents (5.3%) specifically referenced DCM. Fifty percent were operation-specific, most commonly ACDF (23.7%).

**Table 3:** Patient Information Document summary results (N = 38).

		Frequency	%
UK Region	England – South	17	44.7
	England – North	12	31.6
	England – Midlands	5	13.2
	Scotland	3	7.9
	Wales	1	2.6
	Northern Ireland	0	0.0
Publication Date	2018 or before	4	10.5
	2019–2021	6	15.8
	2022–24	26	68.4
	Not stated	2	5.3
DCM Specific	Yes	2	5.3
Operation Specific	Yes	19	50.0
Operation Type	ACDF	9	23.7
	CDR	3	7.9
	Fusion	3	7.9
	Decompression	2	5.3
	Laminectomy	1	2.6
	Anterior discectomy	1	2.6
Background Information	Pathology Information	19	50.0
	Anatomy Information	15	39.5
Pre-Operative Recommendations	Expectation management on outcome	8	21.1
	Smoking cessation	7	18.4
	General physical activity recommendation	3	7.9
	Weight loss advice	3	7.9
	Medications	3	7.9
	Exercise	3	7.9
	Diet	1	2.6
	Reduced alcohol intake	1	2.6
	Social activities	1	2.6
Operative Information	Surgery Information	27	71.1
	Risks of complications	14	36.8
	Post-operative analgesia	11	29.0
	Anaesthetic Information	8	21.1
Logistical Information	Inpatient physiotherapy	17	44.7
	Expected length of stay	16	42.1
	Inpatient care	12	31.6
	Timing, transport, processes	10	26.3
Post-Operative Information	Return to driving	29	76.3
	Return to work	26	68.4
	Pacing and behaviour modification	24	63.2
	Post-discharge expectations	18	47.4
	Social pursuits	18	47.4
	Wound care	18	47.4
	Monitoring complications	18	47.4
	Exercises	17	44.7
	Pain management	16	42.1
	Expectations on throat discomfort and swallowing difficulties	11	29.0
	Advice on sexual intercourse	9	23.7
	Sleeping advice	8	21.1
	Diet	5	13.2
	SaLT advice	2	5.3
	Carer advice	2	5.3
Information on post-hospital care	Outpatient (surgical) appointment details	22	57.9
	Signposting to other forms of advice/information	16	42.1
	Physiotherapy referral	9	23.7

*ACDF* anterior cervical decompression and fusion; *CDR* cervical disc replacement; *DCM* degenerative cervical myelopathy; *SaLT* speech and language therapy; *UK* United Kingdom.

Information most frequently presented in PIDs was on the pathology (50.0%), with over a third presenting information on cervical spine anatomy (39.5%). A range of pre-operative recommendations were provided across the PID. These most frequently provided information on expected post-operative outcomes (21.1%) and advice on smoking cessation (18.4%), with three PID offering information on medications, weight loss, increasing general physical activity and specific exercises (7.9% each).

Patient information documents more frequently offered information on post-operative care and advice compared to pre-operative guidance. This was mostly around information on the operation (71.1%), monitoring risks of complications (36.8%), information on post-operative analgesia (29.0%) and anaesthetic information (21.1%). Identified PID also provided information in what patients were to expect during their inpatient-stay, most frequently on expected physiotherapy (44.7%) and overall care provision (31.6%), expected hospital length of stay (42.1%) and logistical information on timing of arrival and operation, how to get to the hospital and what to expect during an inpatient stay (26.3%).

As illustrated in Table 3, a range of post-operative information was provided across the PID. This was most frequently guidance on return to normal activities such as driving (76.3%), work (68.4%) and social pursuits (47.4%). Recovery guidance was also offered on pacing and behaviour modification (63.2%), wound care advice (47.4%), post-operative shoulder and neck exercises (44.7%) and pain management advice and skills (42.1%). Information provision pertaining to dietary adaptations (13.2%) and the role of speech and language therapy (5.3%) and sleeping advice (21.1%) or guidance on sexual intercourse (23.7%) was less frequently offered.

Whilst over half of PID provided information on when routine surgical follow-up appointments were expected (57.9%), only 24% provided information on whether out-patient physiotherapy appointments would be arranged, with all but one PID outlining this would be if needed at the hospital team’s judgement, rather than routine provision.

## Discussion

The findings of this study highlight the evidence on-which the rehabilitation of people following DCM surgery is poor, with no robust RCTs. The available evidence on post-operative rehabilitation is limited in quality, with studies identified assessing the effect of prehabilitation before DCM surgery. Current NHS patient information before and after DCM surgery focuses on preparation of patient expectedness and awareness of risk, harms and potential complications. Whilst education and guidance is commonly offered on returning to work, driving and normal activities of daily living, there was less frequent information offered on post-operative exercise or approaches to improve health and lifestyle pre-operatively. The PID review indicated that most centres considered post-operative rehabilitation discretionary. Based on current literature and patient-facing materials, there is uncertainty on what should be offered to patients following DCM in respect to prehabilitation or post-operative rehabilitation. Accordingly, this paper acts to inform surgeons, rehabilitation doctors, physiotherapists, occupational therapists and researchers on the status of the current clinical evidence-base on prehabilitation and post-operative rehabilitation for people undergoing DCM surgery. It also highlights, with the AO Spine RECODE-DCM research priority setting exercise [2], an important research priority to researchers, to understand the current limitations on-which this evidence-base is underpinned by, and to stimulate further research in the area.

A principal objective of DCM surgery is to cease the progression of neurological impairment and symptoms [27]. Whilst some evidence suggests that functional outcomes and symptoms can improve [3, 4], it is not clear if this improvement can be enhanced through the provision of prehabilitation interventions or post-operative rehabilitation strategies. Studies identified in the systematic review such as Lorentz et al. [22], Tamai et al. [24] and Catz et al. [20] suggest potential benefits offered to outpatient and in-patient rehabilitation. However, in the absence of a clear comparator [20] or detailed explanation on the constituents of rehabilitation [22], the evidence-base to determine effectiveness on physiotherapy is not sufficiently robust. All studies failed to report the impact of tailoring and personalisation in rehabilitation and how this was assessed to determine intervention fidelity. This is particularly important given the variability in potential interventions from exercise programmes to social functional rehabilitation as more occupational-based interventions, both of which demonstrated promise [24]. Given the heterogeneity in motor and sensory deficit and the spectrum of aspirations which patients have post-operatively, tailoring rehabilitation interventions to patient-need is imperative. This also supports the recent SPINE20 international recommendations for interventions which acknowledge diverse patient needs, to adopt and integrate evidence-based rehabilitation interventions which serve all members of the community [28]. The current evidence is ‘blunt’ in understanding what is and what could be offered to patients following DCM surgery. Furthermore, given the persistence in long-term disability post-DCM surgery [7] and low return to work rates [6], identifying a potentially effective rehabilitation model to enhance clinical outcomes and reduce long-term health service requirements and societal costs is advantageous not only to patients and their families, but also to health and social care systems. This provides a strong rationale for developing and robustly evaluating the clinical and cost-effectiveness of a personalised rehabilitation model for people undergoing DCM surgery.

The systematic review identified that no studies have assessed the effectiveness of prehabilitation for people awaiting DCM. Similar to DCM, in other surgical procedures, people can experience significant pain, loss of movement and reduced function post-operatively during recovery [7, 8]. This may be enhanced or prolonged post-discharge, due to uncertainty on what to do to promote recovery or how to manage symptoms due to limited education or awareness on self-management. Increasing knowledge and confidence to empower people with a prehabilitation programme may optimise post-operative recovery [8]. The findings from the PID review identified limited evidence is offered on self-management skills and strategies. Skills such as pain management guidance, exercise, behaviour modification or pacing activities once discharge were infrequently and inconsistently reported. Whilst this offers an insight into variation in practice, we were unable to determine through this study’s methods what current, face-to-face practice is in the NHS or abroad on prehabilitation or post-operative education provision for patients or their carers. Further consideration on both the needs of patients and families in the pre-operative and post-operative stages of DCM surgery, and how these may form the basis for interventions to support these people, is an important next step in advancing DCM care.

This systematic review and scoping assessment of PID presented with both strengths and limitations. Strengths included a comprehensive and robust literature search of the current, global, evidence on prehabilitation and post-operative rehabilitation for people undergoing DCM surgery. Whilst the systematic review was international, the PID review was limited to NHS providers in the UK, thereby attempting to offer a national picture of online materials which could be searched by the public, in preparation for and after DCM surgery. Whilst the UK-based approach is a representative exemplar for similar work to be performed internationally, it can only make inferences on UK practice. Furthermore, whilst several large UK centres based in London, Liverpool, Cambridge and Sheffield (for example) provided online materials, there was limited reporting of centres from Wales, Scotland and none from Northern Ireland, thereby limiting the generalisability for review materials in the UK context. Similarly, the databases searched for the literature review were those of largely English-speaking countries. Therefore, it remains unclear whether other countries, which may have different healthcare provision and publish literature not deposited on western databases such as EMBASE or MEDLINE, offer on prehabilitation or post-operative rehabilitation programmes. Further scoping with other quantitative or qualitative methods to further understand management strategies around DCM surgery may complement these findings and offer a better understanding of current practices. Finally, whilst not a limitation of our methodology, the evidence-base underpinning rehabilitation post-DCM surgery has a limited follow-up period. Previous literature has recommended patients are followed-up for a minimum of 12- to 24-month post-operatively based on typical recovery trajectories [29]. Accordingly, future researchers should consider adequate follow-up, to determine the efficacy of different rehabilitation approaches for this population.

## Conclusions

While rehabilitation is a gold-standard in spinal cord injury care, the PID review indicates it is considered optional for DCM. This is likely due to a limited (or non-existent with respect to prehabilitation) evidence-base. Although currently based on low-quality evidence, post-operative rehabilitation following DCM surgery appears to show potential across a range of outcome domains such as function, pain and quality of life. Finding ways to enhance the benefits of surgery and reducing long-term disability and healthcare costs are global priorities. Robust clinical trials are imperative to establish effective rehabilitation strategies for DCM.

### Data Sharing

The data that support the findings of this study may be available from the corresponding author (TS) upon reasonable request.

## Supplementary information

Supplementary File
