# Supplementary material for: Rehabilitation for degenerative cervical myelopathy: systematic review and scoping review of UK patient information
Source: Spinal Cord. 2025 Jul 23;63(9):482–91. doi: 10.1038/s41393-025-01110-z (PMC12413311; doi:10.1038/s41393-025-01110-z)
Supplement: Supplementary file 1 — Supplementary File [file 41393_2025_1110_MOESM1_ESM.docx]

**Supplementary File 1:** Search Strategy used for EMBASE (19 May 2025)

1. exp Neck/
2. neck.mp.
3. exp Cervical Spine/
4. exp Cervical Spondylosis/
5. or/1-4
6. exp Spinal Cord Compression/
7. myelopathy.mp.
8. degenerative cervical myelopathy.mp.
9. spinal stenosis.mp.
10. exp Myeloradiculopathy/
11. radiculomyelopathy.mp.
12. cervical spondylotic myelopathy.mp.
13. ossification of the posterior longitudinal ligament.mp
14. ligament calcinosis.mp.
15. OPLL.ti.ab.
16. or/6-15
17. exp posterior longitudinal ligament/
18. exp ossification/
19. ossifi*.ti.ab.
20. or/17-19
21. and/16,20
22. exp Surgery/
23. surgery.mp.
24. surgical.mp.
25. laminectory.mp.
26. decompression.mp.
27. ACDF.mp.
28. Disc replacement.mp.
29. Disc arthroplasty.mp.
30. Fusion.mp.
31. or/22-30
32. and/21,31
33. and/5,32
34. exp Physiotherapy/
35. exp Rehabilitation/
36. exp Exercise/
37. physical therapy.mp.
38. exercise.mp.
39. rehabilitation.mp.
40. prehab.mp.
41. pre-habilitation.mp.
42. physiotherapy.mp.
43. physical therapy.mp.
44. or/34-43
45. and/33,44
46. Nonhuman/
47. exp Animal/
48. Animal Experiment/
49. or/46-48
50. 45 not 49

**Supplementary File 2:** Search strategy for Google search of Patient Information Documents (01 November 2024)

1. rehabilitation AND degenerative cervical myelopathy AND nhs
2. physiotherapy, degenerative cervical myelopathy, nhs
3. prehabilitation, degenerative cervical myelopathy, nhs
4. patient information, degenerative cervical myelopathy, nhs
5. rehabilitation, cervical surgery, nhs
6. physiotherapy, cervical surgery, nhs
7. prehabilitation, cervical surgery, nhs
8. patient information, cervical surgery, nhs
9. rehabilitation, neck operation, nhs
10. physiotherapy, neck operation, nhs
11. prehabilitation, neck operation, nhs
12. patient information, neck operation, nhs
